# MHC-Optimized Peptide Scaffold for Improved Antigen Presentation and Anti-Tumor Response

**DOI:** 10.3389/fimmu.2021.769799

**Published:** 2021-10-20

**Authors:** Maria Tagliamonte, Angela Mauriello, Beatrice Cavalluzzo, Concetta Ragone, Carmen Manolio, Antonio Luciano, Antonio Barbieri, Giuseppe Palma, Giosuè Scognamiglio, Annabella Di Mauro, Maurizio Di Bonito, Maria Lina Tornesello, Franco M. Buonaguro, Luigi Vitagliano, Andrea Caporale, Menotti Ruvo, Luigi Buonaguro

**Affiliations:** ^1^ Innovative Immunological Models Lab, Istituto Nazionale Tumori - Istituto di Ricovero e Cura a Carattere Scientifico (IRCCS) - “Fond G. Pascale”, Naples, Italy; ^2^ Animal Facility, Istituto Nazionale Tumori - Istituto di Ricovero e Cura a Carattere Scientifico (IRCCS) - “Fond G. Pascale”, Naples, Italy; ^3^ Pathology Unit, Istituto Nazionale Tumori - Istituto di Ricovero e Cura a Carattere Scientifico (IRCCS) - “Fond G. Pascale”, Naples, Italy; ^4^ Molecular Biology and Viral Oncogenesis, Istituto Nazionale Tumori - Istituto di Ricovero e Cura a Carattere Scientifico (IRCCS) - “Fond G. Pascale”, Naples, Italy; ^5^ Institute of Biostructures and Bioimaging, Consiglio Nazionale delle Ricerche (CNR), Napoli, Italy

**Keywords:** cancer vaccine, heteroclitic peptides, TAA, major histocompatibility complex I (MHCI), peptide scaffold

## Abstract

Tumor Associated Antigens (TAAs) may suffer from an immunological tolerance due to expression on normal cells. In order to potentiate their immunogenicity, heteroclitic peptides (htcPep) were designed according to prediction algorithms. In particular, specific modifications were introduced in peptide residues facing to TCR. Moreover, a MHC-optimized scaffold was designed for improved antigen presentation to TCR by H-2Db allele. The efficacy of such htcPep was assessed in C57BL/6 mice injected with syngeneic melanoma B16F10 or lung TC1 tumor cell lines, in combination with metronomic chemotherapy and immune checkpoint inhibitors. The immunogenicity of htcPep was significantly stronger than the corresponding wt peptide and the modification involving both MHC and TCR binding residues scored the strongest. In particular, the H-2Db-specific scaffold significantly potentiated the peptides’ immunogenicity and control of tumor growth was comparable to wt peptide in a therapeutic setting. Overall, we demonstrated that modified TAAs show higher immunogenicity compared to wt peptide. In particular, the MHC-optimized scaffold can present different antigen sequences to TCR, retaining the conformational characteristics of the corresponding wt. Cross-reacting CD8^+^ T cells are elicited and efficiently kill tumor cells presenting the wild-type antigen. This novel approach can be of high clinical relevance in cancer vaccine development.

## Introduction

Tumor associated antigens (TAAs) are cellular self-antigens mostly overexpressed in cancer cells with low expression in normal cells. For such reason, they may be subject to both central and peripheral tolerance mechanisms leading to an inefficient immune response. Indeed, TAA-based cancer vaccines are well tolerated and have minimal side effects but have proven to have limited efficacy in clinical trials (1, 2). On the contrary, such a limitation is not observed when using tumor-specific neoantigens (TSAs) which arise from protein mutations and are solely expressed on the tumor cells. However, TSAs are strictly private to each individual cancer patient and their identification is laborious as well as expensive (3).

Recognition of target non-self-antigens by T cells is mediated by interaction between the T cell receptor (TCR) and the peptide-MHC-I complex (pMHC). In such interaction, the peptide is fastened to the MHC-I groove through the amino acid residues at the “anchor positions”, exposing the “TCR-binding” residues to the TCR. The deciphering of the different MHC-I binding motifs has allowed the definition of the preferred peptide amino acid residues at the anchor positions for each HLA allele. In particular, the vast majority of data have been generated for the HLA-A*02:01, showing that the xLxxxxxx^V^/_L_ is the most efficient binding motif to fit into the HLA groove (4–7). However, most of the natural HLA-A*0201-restricted cancer-derived epitopes do not show the best-fitting binding motif (https://caped.icp.ucl.ac.be/) and htcPep have been designed with an enhanced stability of the pMHC-I complex as well as an increased immunogenicity of the bound peptide (8–11). Nevertheless, mutations introduced in the anchor-positions may generate a perturbation in the epitope’s conformation resulting in structural changes to the TCR-binding residues and affecting the T-cell recognition (12).

An alternative approach for improving the immunogenicity of natural TAAs is to generate htcPep keeping the same “anchor residues” and mutating the TCR-binding residues only. The aim is to obtain an epitope sufficiently different from the natural wild-type peptide presented by the cancer cells in order to break the immunological tolerance and induce a more potent CD8^+^ T cell response. However, such a difference should not be too extensive to retain the capability of eliciting cross-reactive T cell response able to recognize the wt peptide and kill the presenting tumor cells (13). Indeed, the low affinity between the TCR and the pMHC allows the TCR to cross-react with multiple pMHCs (14–16).

Recently, we demonstrated that modified heteroclitic TAAs, Trp2 and HPV-E7 peptides, expressed by mouse melanoma B16F10 and TC1 tumor cell lines, respectively, can be improved by changing even one single side chain of the TCR-binding residues (i.e. p4), achieving higher affinity to MHC-I molecule (17).

In the present study, such heteroclitic forms of Trp2 and HPV-E7 peptides were immunologically validated in a mouse model. Moreover, the most effective heteroclitic peptide was further modified, generating a H-2Db-optimized scaffold by introducing at each “anchor” position the best fitting residue. MHC binding as well as induction of anti-tumor immune response were experimentally assessed in two experimental models, showing the improved efficacy of such scaffold strategy for developing a cancer vaccine.

## Materials and Methods

### Cell Line and Mice

C57BL/6 (H-2b MHC) female mice, 8 weeks old, were purchased from Harlan (Udine, Italy). All animals were housed at the Animal Facility of the Istituto Nazionale Tumori “Pascale” (Naples, Italy). Mice were housed in number of 2-3 per cage and maintained in a conventional facility on a 12 hrs light:12 hrs dark cycle (lights on at 7:00 a.m.) in a temperature-controlled room (22 ± 2°C) and with food and water ad libitum at all times. The experimental protocols were in compliance with the European Communities Council directive (86/609/EEC).

Mouse melanoma B16F10 (ATCC, CRL-6323) cells were cultured in DMEM medium supplemented with 10% heat inactivated FBS, 100 U/ml penicillin and 100 mg/ml streptomycin (Invitrogen, Carlsbad, CA) at 37°C with 5% CO2. Cells were tested for mycoplasma before inoculation in mice (ATCC^®^, 30-1012K™). Mouse lung TC-1 tumor cells expressing HPV16 E7 protein (ATCC, CRL-2493), were cultured in RPMI medium supplemented with 10% heat inactivated FBS, 100 U/ml penicillin and 100 mg/ml streptomycin (Invitrogen, Carlsbad, CA), HEPES 10mM, MEM 0.1 mM and Na Piruvate 1 mM at 37°C with 5% CO2.

### Design and Peptide Synthesis

Heteroclitic peptides (htcPep) with substitution in the p4 residue were described in our previously study (17). An additional htcPep was designed introducing at p4 a non-natural aa residue based on the Tryptophan structure (NAL). Alternatively, a peptide library including 20 peptides was prepared by random introduction of all 20 possible amino acids at position 4 (MIX). Individual htcPep were synthesized at a purity > 95% determined by LC-MS analyses. Lyophilized powder was dissolved in dimethylsulfoxide (DMSO; Sigma-Aldrich), diluted in phosphate-buffered saline (1× PBS; Gibco Life Technologies) and stored at − 80°C until use.

### Drugs Administration

Cyclophosphamide (CTX) (Endoxan^®^, Baxter) (10 mg/Kg) and Paclitaxel (PTX) (Taxol^®^, BMS) (5mg/Kg) diluted with phosphate-buffered saline (PBS) were administered *via* intraperitoneal injection (i.p.). The dose was extrapolated to human equivalent dose (HED) according to Reagan-Shaw et al. (18). Metronomic Chemotherapy was weekly administered one day before the vaccine administration until the end of the experiment. An anti-mouse PD-1 MAb (BioXCell, West Lebanon, NH USA) was used as checkpoint inhibitor (ICI) and weekly administered *via* intraperitoneal injection (i.p.) at a dose of 100μg.

### Immunizations Protocol for Biological Validation of Cross-Reactivity Between wt and htcPep

C57BL/6 mice were immunized with the different htcPep. In particular, each group of animals (6 for each group) were injected by sub-cutaneous (s.c.) route with 100 µg of WT, INC (mix of selected htcPep), NAL or MIX peptides (peptide library) respectively, emulsified with 50 μg of Polyinosinic:polycytidylic acid [poly(I:C); InvivoGen] adjuvant formulated in PBS (200ul total volume). Each group received weekly the peptide vaccine in combination with metronomic chemotherapy (MCT) and ICI. One group was treated only with MCT and ICI (MCT + ICI group). Control mice were treated with endotoxin-free phosphate-buffered saline (PBS). Splenocytes were re-stimulated *ex-vivo* O/N with the different peptides in an IFN-γ EliSpot assay.

### Animal Experiments

B16F10 and TC1 cells were harvested in exponential growth phase by trypsinization and washed twice with ice-cold PBS. C57BL/6 mice were subcutaneously injected with 5x10^4^ and 1x10^5^ cells/mouse of B16F10 or TC1 respectively on the right back flank. The tumor size was measured and documented every two days with a caliper, starting on day 7, and calculated using the formula (AxB^2^)/2 (A as the largest and B is the smallest diameter of tumor). Tumor growth was documented as mean tumor size with standard error. To record the survival of the tumor-bearing mice, either natural death or a tumor diameter greater than 1500 mm^3^ leading to death was counted as death.

### Therapeutic Immunization Experiment

B16F10 as well as TC1 cell lines were subcutaneously (s.c.) injected into the left flank of all mice as above described. When the tumor diameter reached 4-6 mm, C57BL/6 mice were randomly divided into six groups and immunized with the vaccine once a week. In particular, each mouse of each experimental group (6 or 8 for each group) was immunized with 100 ug of peptide vaccine both for single peptides (WT and NAL) and for htcPeps mixes (INC and MIX) in total, emulsified with 50 μg of Polyinosinic:polycytidylic acid [poly(I:C); InvivoGen] adjuvant formulated in PBS (200ul total volume). Each group received the peptide vaccine in combination with metronomic chemotherapy (MCT) and ICI. One group was treated only with MCT and ICI (MCT + ICI group). Control mice were treated with endotoxin-free phosphate-buffered saline (PBS) (**Supplementary Figures 1, 7**).

### IFN-γ ELISpot Assay

ELISPOT was performed according to BD Biosciences manufacturer instructions (BD ELISPOT Mouse IFN-γ ELISPOT Set cod. 551083). 5x10^5^ splenocytes or 2 x10^5^ tumor infiltrated lymphocytes (TIL) were counted and plated in each well. In brief, tumor biopsies were cut into small fragments ∼2–3 mm in length and subjected to a commercial mechanical/enzymatic dissociation system (GentleMACS, Miltenyi Biotec, Bergish Gladbach, Germany). After disaggregation, the single cell suspension was passed through 70-*μ*m strainers. TIL were isolated from dissociated tumors by CD45 (TIL) MicroBeads (Miltenyi biotec), for positive selection of CD45-specific TIL. Enriched TILs were cultured in complete media (RPMI 1640 (Lonza) supplemented with 10% fetal calf serum (FCS), 1% glutamine, 100 IU ml^−1^ penicillin, 100 *μ*g ml^−1^ streptomycin (all from Life Technologies, Paisley, UK).

Both splenocytes and TILs were stimulated with 10ug/ml of single or peptide pool used for the immunization and incubated for 24-26h. As negative and positive control, peptide diluents PBS and 5ug/ml of phorbol myristate acetate (PMA, Sigma-Aldrich) were used respectively. The plates were read with an AID EliSpot Reader Systems (AID GmbH, Strassberg, Germany). The results were calculated as spot forming counts as a mean of a duplicate count from the specific antigen stimulation minus the negative control.

### Cell-Mediated Cytotoxicity Assay

Measurements of cytotoxic T-cell activity were performed on spleen resected from each mouse involved in each experiment. TC1 and B16F10 cell lines were used as target cells and 7-AAD/CFSE Cell-Mediated Cytotoxicity Assay Kit was used to assess the cell-mediated cytotoxicity. Briefly, CFSE-based cytotoxity assay was performed as previously described with slightly modification (19). Target cells were labeled with 5 μM CFSE (BD Biosciences) for 10 min and then cocultured with isolated spleens at 37°C for 4 h, at E: T ratio of 1:1, 5:1, 10:1 and 50:1. After the coculture, 1 μg/mL propidium iodide (PI, BD Biosciences) was added for assigning the ratio of cell death, and the samples were analyzed by flow cytometry.

Prediction and Design of a H-2Db-specific epitope scaffold.

The sequence of E7 and Trp2 wild type protein was used to predict epitopes binding to the mouse H-2Db allele by the NetMHCpan 4.1. The same analysis was performed using 20 different random proteins. A total number of 250 predicted strong binders were aligned from position 1 to position 9 and analyzed by the online Seq2Logo - 2.0 (https://services.healthtech.dtu.dk/service.php?Seq2Logo-2.0). The most frequent amino acid residues found at the anchor positions (p2,p3,p5,p9) were selected to design the H-2Db best fitting motif and generate the epitope scaffold for optimal presentation of the TCR-binding residues.

### Peptide Binding Affinity Assays

Peptide binding affinity to H-2Db molecule was performed for each selected peptide. TAP-deficient RMA-S cells were cultured for 24 hour at 26°C to accumulate empty MHC-I molecules on the cell surface (20). Cells were washed in serum-free medium and incubated at 3x10^5^ cells/well with peptides (10 μM) for 4 h at 37°C with 5% CO_2_. Cells were washed and stained for H-2Db as described above and analyzed by flow cytometry. All the experiments were performed in triplicate.

### Molecular Docking

Structural data available for H-2Db/HPV-E7 as well as for H-2Db/Trp2 complexes H-2Db was not found in the Protein Data Bank (PDB) (https://www.rcsb.org/). Therefore, the PDB entry 1FG2 corresponding to a complex of H-2D^b^ with a peptide with sequence KAVYNFATC was selected for docking analyses (21). The molecular modelling and docking analyses were performed by PyMOL (ver. 1.8.6.2) and the ICM-Browser (ver. 3.8; MolSoft LLC) molecular graphics systems.

### Immunohistochemistry

Formalin-Fixed Paraffin-Embedded (FFPE) sections were stained with CD3+, FoxP3 and Granzime B antibodies manually. Visualization of the antibody–antigen reaction was visualized by anti-alkaline phosphatase methods using Vulcan fast red (Biocare Medical FR805S) as the chromogenic substrate. Finally, sections were weakly counterstained with hematoxylin and mounted.

The manufacturers and condition use of primary antibodies are summarized in **Table 1**.

**Table 1:** Manufacturers and condition use of primary antibodies.

	Host	Clone	Location	Epitope Retrieval	Dilution (μg/mL)	Diluent antibody	Code (Company)
**CD3**	Rabbit	SP7	Cytoplasmic	PH6	1:150	Dako AR9352	ab16669 (Abcam)
**FoxP3**	Rabbit	mAbcam 22510	Nuclear	PH8	1:250	Dako AR9352	ab22510 (Abcam)
**GranzimeB**	Rabbit	Polyclonal	Cytoplasmic	PH8	1:200	Dako AR9352	ab4059 (Abcam)

Lymphocyte densities (cells/mm2) were quantified in the tumor (CT) and invasive margin (IM) by digital pathology. The slides were scanned with Aperio AT2 (Leica) and the areas (CT, IM) were selected by an experienced pathologist (MDB). Finally, the acquired images were quantified using QuPath 0.2.3.

To evaluate the balance between cytotoxic and immune suppressive activity of B16 tumor model a formula considering the numerical difference between the GrzB+ cells (cytotoxic cells) and the FOXP3+ cells (immunosuppressive cells), on total CD3+ cells, was applied. The formula is schematized as:


Ratio=(# of GRZB−# of FOXP3)/# of CD3.

A negative ratio result meant an immunosuppressive environment, while a positive ratio was indicative of an active cytotoxic environment.

Finally, tumor necrosis rates were evaluated for each tumor lesion. The slides were stained with hematoxylin and eosin and then subjected to reading and determination of areas of necrosis. For percentage of necrosis through the tumor mass, was used the following formula: …


%necrosis area: (necrosis area/total tumoral mass area)∗100

### Statistical Analysis

Comparison between individual data points were performed with the unpaired two-sided Student’s *t*-test and ANOVA, as appropriate. Normally distributed data were represented as mean ± S.E.M. Two-way ANOVA and Bonferroni *post-hoc* analysis were used to examine the significance of differences among groups. All P values were two-tailed and considered significant if less than 0.05.

## Results

### Biological Validation of Cross-Reactivity Between wt and htcPep

We have recently described htcPep for Trp2 and HPV-E7 antigens modified in the TCR – binding position 4 with different aa residues showing diverse structural conformation as well as binding affinity to H-2Db allele as compared to wt (17). Additional htcPep were designed for the Trp2 and HPV-E7 peptides introducing at p4 a non-natural aa residue based on the Tryptophan (TRP, W) structure (NAL). Moreover, a peptide library including 20 peptides was prepared by random introduction of all 20 possible amino acids at position 4 (MIX).

To assess the cross-reactive T cell response of all these htcPep, C57BL/6 mice were immunized and the immunological cross-reactivity was evaluated in an IFN-γ EliSpot assay.

**Figure 1:**
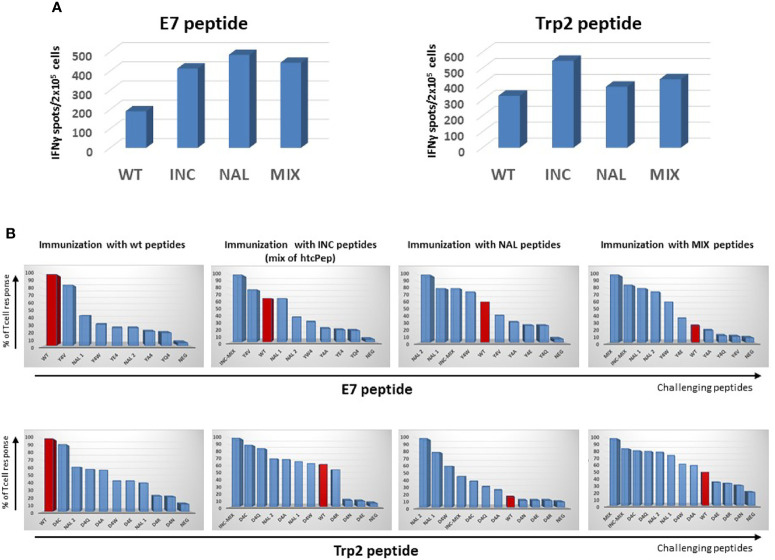
Cross-reactive T cell response. C57BL/6 mice were immunized with indicated peptides and an IFN-γ EliSpot assay was performed re-stimulating *ex vivo* splenocytes with the indicated challenging peptides. **(A)** Absolute immune responses elicited by wild type and heteroclitic peptides. **(B)** Percentage of T cell response compared to the one observed by re-stimulation with the same peptide used in the immunization (= 100).

Animals immunized with the wt peptides showed a significant T cell cross-reactivity with htcPep, and the best scoring was the Y4V (90% of reactivity), for the E7 peptide, and the D4C (93% of reactivity), for the Trp2 peptide (**Figure 1**).

As further confirmation, when animals were immunized with the INC peptides (mix of htcPep), T cells cross-reacted with the corresponding wt peptide although at lower level (68% and 63% of reactivity for the HPV-E7 and Trp2, respectively). Consistently, when animals were immunized with NAL peptide, the strongest T cells cross-reactivity was observed with the htcPep with a Tryptophan substitution at position 4 (Y4W for the E7 and D4W for the Trp2). In the latter case, the cross reactivity with the wt peptide was significantly lower, especially for the Trp2 epitope (23% of reactivity) (**Figure 1**).

### Effect on Tumor Growth of Therapeutic Immunization With htcPep

The immunogenicity and the induction of the anti-tumor T cell immunity by the htcPep was assessed in a therapeutic setting in C57BL/6 mice injected with either E7-expressing TC1 or Trp2-expressing B16F10 cell lines (**Supplementary Figure 1**).

Animals were monitored during the whole protocol and no toxicity was observed, showing a good general status without any significant weight loss (data not shown). Tumor volume in all the different groups was compared when the tumor reached the cutoff of 1600 mm^3^ in the last animal of the control group.

**Figure 2:**
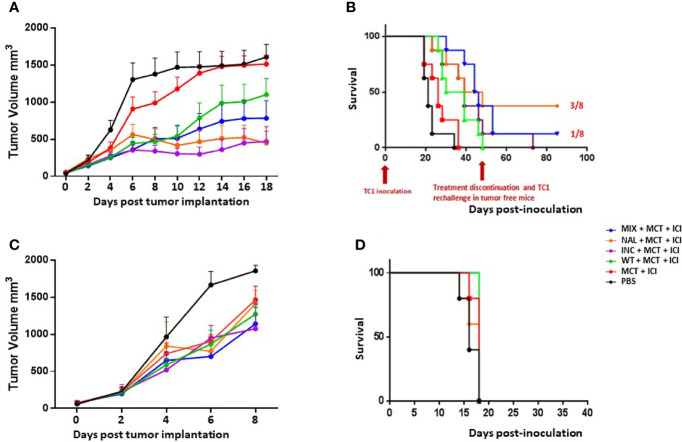
Cancer vaccine efficacy in therapeutic setting. C57BL/6 mice were administered with TC1 or B16F10 tumor cell lines. When palpable, indicated treatments were initiated. TC1 experimental model: **(A)** Tumor growth; **(B)** Keplein Mayer curve. B16F10 experimental model: **(C)** Tumor growth; **(D)** Keplein Mayer curve.

The results showed that all the combinatorial strategies (MCT+ICI+PEP) induced a significant delay in tumor growth at least in TC1 tumor model. In particular, all the htcPep were more effective than the wt, and the best result was achieved in the experimental groups immunized with the NAL peptide and the INC (mix of selected htcPep) (**Figure 2A** and **Supplementary Figure 2**). The results were confirmed by significant improvement in the survival. Treatment was discontinued at day 48, when animals were still tumor-free in the htcPep experimental groups only. The latter were re-challenge with the same tumor cells and tumor growth was completely controlled in 3/8 in the NAL group and 1/8 in the MIX group (*p* < 0.01) (**Figure 2B**). Results in the B16F10 tumor model were drastically less effective. Overall, tumor growth was much faster than the TC1 and the cutoff was reached in the last animal of the control group at day 8 (**Figure 2C**). Neither the wt nor the htcPep provided a significant improvement in the delay of tumor growth compared to the combination of MCT+ICI (**Figure 2C** and **Supplementary Figure 3**) and this result reflected also in the survival curves (**Figure 2D**).

### Immunological Correlates in the Therapeutic Setting

**Figure 3:**
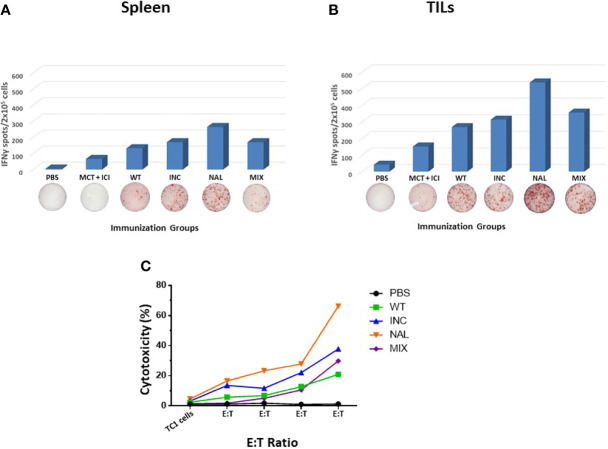
Immune correlates in the therapeutic experimental model. Spleens **(A)** and TILs **(B)** from sacrificed animals in each HPV-E7 immunization groups were evaluated in an IFNγ EliSpot assay after re-challenge with wt epitope. **(C)** Cytotoxicity assay was assessed using TC1 cells as target cells at the indicated target:effector (T:E) ratios. Each peptide immunization was performed with the MCT + ICI combination.

In order to assess the immune correlates of the results in the therapeutic setting, spleens and Tumor infiltrating lymphocytes (TILs) were collected from immunized animals when sacrificed. TILs were not evaluable for the B16F10 model given the immunological “cold” pattern and the extremely limited number of cells isolated from the tumor lesions. The IFNγ EliSpot assay performed with spleens from immunized animals showed an increased T cell response against the wt peptide in the htcPep groups, which reached a statistical significance in the NAL group. Such effect was much greater in TILs, strongly supporting the biological effect on the tumor growth and overall survival (**Figures 3A, B**). The results were further confirmed by a cytotoxicity assay in which spleens from the NAL group showed the highest cytotoxicity against the TC1 cells (**Figure 3C**). On the contrary, results obtained in the B16F10 were of much lower magnitude and the NAL group showed a T cell reactivity even lower than the wt group (data not shown).

### Prediction and Design of H-2Db-Specific Epitope Scaffold

In order to verify whether an *in silico* predicted sequence could increase the binding affinity of the wt epitopes to the H-2Db molecule and improve their immunogenicity, an epitope scaffold was generated, selecting residues at the “anchor positions” (p2, p3, p5, p9) predicted to provide the best binding to the MHC molecule.

To this aim, a sequence logo was generated for construction and visualization of amino acid binding motifs and sequence profiles with sequence weighting (22). More than 250 peptides strong binders to the H-2Db molecule were used to build the logo. The analysis of relative residue frequencies at each position of the epitopes, showed that specific residues are significantly preferred at anchor positions (p2, p3, p5, p9) binding the MHC H-2Db molecule (**Supplementary Figure 4**). In particular, the leucine (L) residue is the most frequent in p3 and p9, while the alanine (A) residue is the most frequent in p2 and the asparagine (N) residue is the most frequent in p5. This finding suggests that the hydrophobic side chain of L favors a good anchorage of the peptide to the H-2Db binding cavity. Based on these results, an epitope scaffold was generated for both HPV-E7 and Trp2 with the sequence xALxNxxxL. In the E7 scaffold, the wt p4 residue was also changed with the NAL residue; while, in the Trp2 scaffold, the wt p4 residue was changed (D4Q and D4C) according to the previous evidence of improved affinity to MHC (**Supplementary Figures 5A, B**).

Prediction of binding to the H-2Db MHC-I was obtained for the scaffold peptides with the prediction algorithm NetMHCpan4.1. The modification in the anchor positions had significant impact on the predicted binding affinity, but it was more dramatic for the Trp2. Indeed, the Trp2 scaffold (Trp2-scaff) shows a 356.6-fold increase in the binding affinity to the H-2Db compared to the Trp2 wt (19.6 vs. 6990 nM). Moreover, when the Gln or Cys substitutions in P4 were introduced (TRP2-scaff/D4C and TRP2-scaff/D4Q) the binding affinity was further improved, reaching a thousand-fold increase over the Trp2 wt (6.6 nM and 6.4 nM, respectively) (**Supplementary Figure 5D**). Similar results were obtained for the HPV-E7 peptide, although the fold increase in the binding affinity to the H-2Db was less noticeable because the wt sequence already shows optimal residues at p2 and p5 anchor positions and is a strong binder (99.7 nM). Nevertheless, a 17.2-fold increase in the binding affinity of the HPV-E7 scaffold (E7-scaff) compared to the HPV-E7 wt was observed (5.8 vs. 99.7 nM) (**Supplementary Figure 5C**). Prediction of binding affinity was not possible with the E7-scaffold including the non-natural NAL amino acid at p4.

### Structure Modelling and Molecular Docking

**Figure 4:**
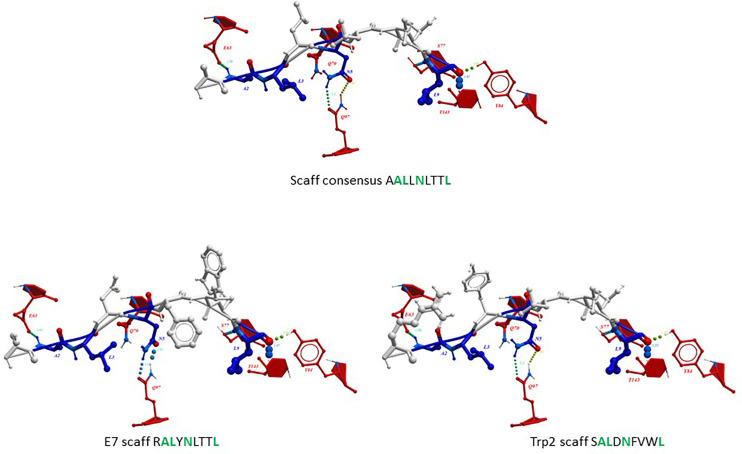
Predicted conformation of scaffolds. The conformation of the HPV-E7 and Trp2 scaffolds bound to the H2-Db molecule are compared to the consensus scaffold. The hydrogen bonds with H-2Db residues are indicated for the anchor positions.

The impact of the amino acid substitutions at the anchor positions in the scaffolds (p2, p3, p5, p9) on the structure of the peptides as well as the interaction with the H-2Db molecule, was assessed by structure modelling and molecular docking comparing with the corresponding wt peptide. In addition, the scaffolds were compared with a hypothetical peptide with the amino acid sequence derived from the most frequent residues at each position as for the sequence logo. The results showed that, regardless the different amino acid residues in the TCR-facing positions (p1, p4, p6, p7, p8), the conformation of the scaffolds and the interaction of the anchor positions with the H-2Db molecule was perfectly retained. Indeed, no changes in the hydrogen bonds between the scaffolds’ anchor positions and the H-2Db residues were observed (**Figure 4**). This confirmed the hypothesis that *in silico* designed scaffolds can represent a universal optimal presentation strategy for a given MHC molecule.

**Figure 5:**
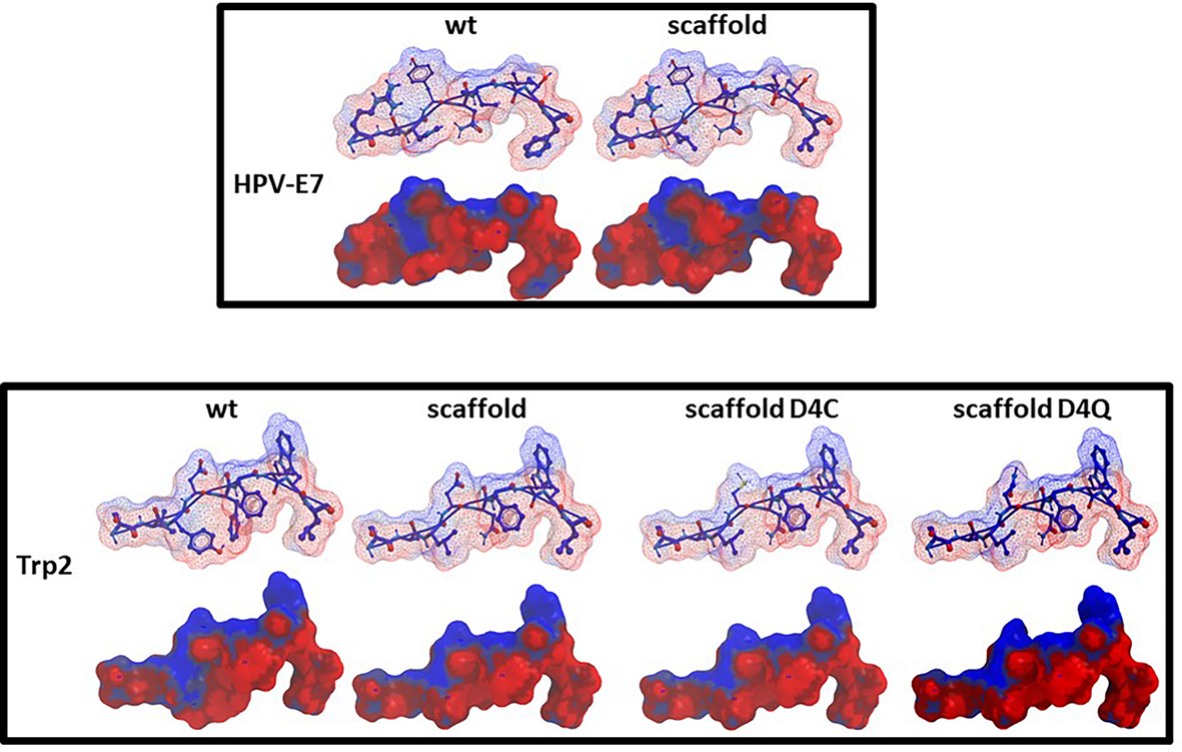
Predicted conformation of scaffold peptides. The conformation of the HPV-E7 and Trp2 scaffold peptides, bound to the H2-Db molecule, is shown compared to the wt sequence. Contact pattern to the H-2Db is indicated in red; contact pattern to TCR is indicated in blue.

Furthermore, the scaffolds’ TCR-facing structure showed highly comparable conformation with the wt epitopes, even when mutations are introduced at p4, supporting the possibility of eliciting a cross-reacting T cell response. Such evidence is quite relevant especially for the Trp2 peptide in which the scaffold differs from the wt in 3/4 residues in the anchor positions and none of the substitutions are conservative (V2A, Y3L, F5N). On the contrary, the E7-scaff differs from the wt in 2/4 residues (H3L and F9L) and the F9L substitution is conservative (**Figure 5**).

### *In Vitro* Analysis of Scaffold Binding Affinity to H-2Db Molecule

The binding affinity of E7-scaff and Trp2-scaff peptides to H-2Db molecule was assessed using the TAP-deficient RMA-S cell line loaded with 10 μM of each peptide. Both scaffolds showed an increased binding to the H-2Db molecule (2 folds for the Trp2-scaff and 4 folds for the E7-scaff) which was further improved when substitutions in p4 were introduced. In particular, for the Trp2-scaff, the highest improvement was observed with the D4C substitution (2-fold increase). Similarly, the substitution in p4 with the NAL non-natural aa induced a 2.7 fold increase in the binding affinity of the E7-scaff to the H-2Db (**Supplementary Figure 6**).

### Effect on Tumor Growth of Therapeutic Immunization With Epitope Scaffolds

The immunogenicity and the induction of the anti-tumor T cell immunity by the epitope scaffolds was assessed in a therapeutic setting in C57BL/6 mice injected with either E7-expressing TC1 or Trp2-expressing B16F10 cell lines (**Supplementary Figure 7**).

Animals were monitored during the whole protocol and no toxicity was observed, showing a good general status without any significant weight loss (data not shown). Tumor volume in all the different groups was compared when the tumor reached the cutoff of 1600 mm^3^ in the last animal of the control group.

**Figure 6:**
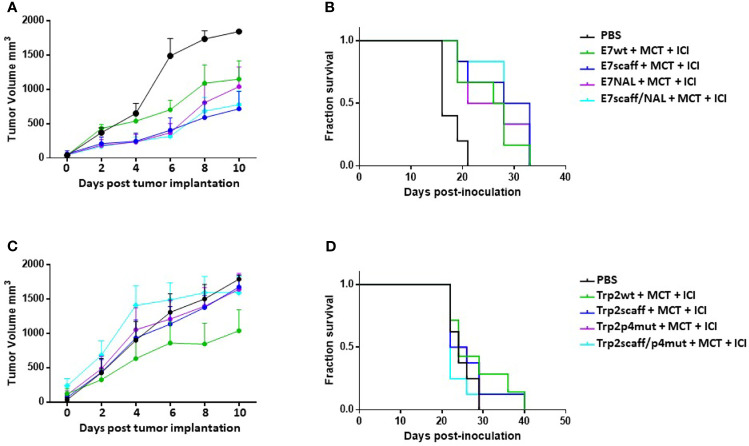
Cancer vaccine efficacy of scaffolds in therapeutic setting. C57BL/6 mice were administered with TC1 or B16F10 tumor cell lines. When palpable, indicated treatments were initiated. TC1 experimental model: **(A)** Tumor growth; **(B)** Keplein Mayer curve. B16F10 experimental model: **(C)** Tumor growth; **(D)** Keplein-Mayer curve.

In the TC1 experimental model, results showed that immunization with the E7-scaff peptides, with or without the NAL modification in p4, was able to control tumor growth. In particular, when compared to the E7 wt, with or without the NAL modification in p4, the efficacy of E7-scaff peptides showed a trend of improvement which did not reach the statistical significance (p = 0.35) (**Figures 6A, B**). On the contrary, in the B16F10 experimental model, immunization with the Trp2-scaff peptides, with or without the modifications in p4, did not improve tumor growth control when compared to Trp2 wt (**Figures 6C, D**). This result apparently contrasted with the observed much higher binding affinity compared to Trp2 wt in the RMA-S cell assay (**Supplementary Figure 6**).

### Immunological Correlates of the Experiments With Epitope Scaffolds

**Figure 7:**
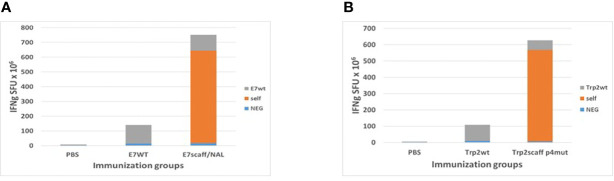
Immune correlates in the immunization experiments with scaffolds. Spleens from sacrificed animals in HPV-E7 **(A)** and Trp2 **(B)** immunization groups were evaluated in an IFNγ EliSpot assay after re-challenge with indicated peptides. Self = same peptide used in the immunization and in the re-challenge assay.

Spleens were collected from immunized animals at the end of the experiment. The IFNγ EliSpot assay confirmed the much higher immunogenicity of both E7-scaff and Trp2-scaff peptides over the corresponding wt peptides. In particular, when the mutation in p4 was introduced, an approximately 6-fold increase in the IFNg SFU was observed (**Figure 7**). Induction by the scaffold peptides of a cross-reactive immune response against the corresponding wt peptides was observed. This was of a significantly lower magnitude, comparable to the immune response elicited by the wt peptide itself (**Figures 7A, B**). Such results confirmed all the *in silico* prediction and *ex vivo* observations showing a superior antigenicity of both scaffolds mutated in the p4 compared to the corresponding wt peptides.

### Evaluation of Tumor-Infiltrating Lymphocytes in B16F10 Tumor Model

In order to elucidate the lack of correlation between the high antigenicity and immunogenicity of the Trp2 scaff peptides and the limited control of B16F10 tumor growth, the Invasive Margin (IM) and Central Tumor (CT) immune contexture was assessed in the tumor lesions resected from animals at sacrifice. In particular, the number of CD3^+^, GrzB^+^ and FOXP3^+^ T cells was evaluated by IHC.

The staining showed infiltrating CD3^+^ T cells in the IM areas of all experimental groups, with an average number ranging from 51.5 (wt scaff) to 103.1 (wt scaff/p4mut). The average number dramatically dropped in the CT areas, except for the wt scaff/p4mut group (81.5).

**Figure 8:**
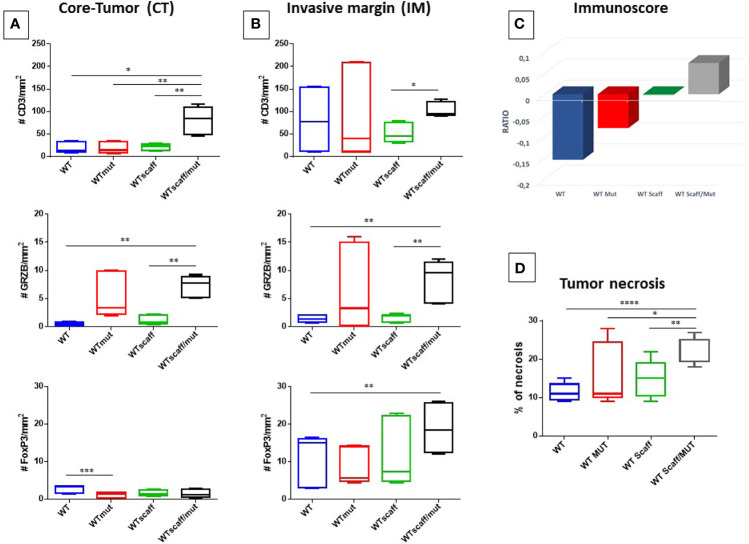
Number of CD3^+^ Granzyme B^+^ and FoxP3^+^ cells infiltrating B16F10 tumors. Cells are represented as mean with standard deviation (SD) in each experimental group. **(A)** CD3^+^, Granzyme B^+^ and FoxP3^+^cells in CT **(A)** and IM **(B)** area by IHC are shown, respectively. **(C)** Cumulative evaluation of the effector cytotoxic GrzB+ and suppressive FOXP3+ T cell on total CD3+ cells. Average standard deviation of the percentage of necrosis in each experimental group is shown **(D)**. Statistical analysis 2way ANOVA: *p < 0.05, **p < 0.005, ***p < 0,001, ****p < 0,0001.

Similarly, although at a lower scale, the average number of FOXP3^+^ T cells is higher in the IM areas of all experimental groups, ranging from 8.1 (wt mut) to 18.8 (wt scaff/p4mut), than in the CT areas, ranging from 1.1 (wt mut) to 2.8 (wt). On the contrary, the average number of GrzB^+^ T cells is much lower and equivalent in the two areas (3.5 CT; 4.4 IM), with the highest average number in the wt scaff/p4mut group (7.2 intra-tumoral; 8.4 peri-tumoral) (**Figures 8A, B**)

The cumulative evaluation of the effector cytotoxic GrzB^+^ and suppressive FOXP3^+^ T cell subtypes in the TME, namely the “immunoscore”, showed that a positive ratio (GrzB^+^/FOXP3^+^) was observed only the wt scaff/p4mut experimental group. This indicates a limited anti-tumor immune effector TME, while all other groups show an immune suppressive phenotype (**Figure 8C** and **Supplementary Figure 8**).

Finally, the percentage of tumor necrosis was evaluated for the different experimental groups. After calculating the necrosis index in the different experimental groups (expressed as the percentage of necrotic areas by total surface estimated by H&E staining), a significant increase of % of necrosis was observed between WT and WT scaff/mut group (10% to 25%) (**Figure 8D** and **Supplementary Figure 9**). However, this high rate of necrosis observed in the WT scaff/mut group is not correlated with tumor regression.

## Conclusions

We have previously described heteroclitic peptides (htcPep) for Trp2 and HPV-E7 antigens modified in the p4 with increased binding affinity to the H-2Db molecule, compared to the wt peptide. Such a prediction, has been experimentally validated by a binding assay in a TAP-deficient RMA-S cell line (17).

In the present study we have designed an additional htcPep with a non-natural aa residue based on the Tryptophan structure (NAL) and generated a peptide library by random introduction of all 20 natural amino acids at position 4 (MIX).

Immunization in a C57BL/6 mouse model confirmed that all htcPep were able to elicit a T cell response cross-reacting with the wt peptide. Further confirmation was provided in a therapeutic vaccination setting, in which T cells elicited by htcPep showed a cross-reactive anti-tumor immunity against tumor cells expressing the wt epitope. Such effect was much more evident in the TC1 than in the B16F10 tumor model which, as previously reported by several groups including ours, is characterized by a poor effector lymphocyte infiltration (“cold tumor”) (23–25).

All such immunological validations support the hypothesis that a modification in the TCR-facing p4 residue not only increases the binding affinity of the wt peptide to the H-2Db molecule but induces a cross-reactive T cell response with anti-tumor activity against tumor cells expressing the wt peptide. Interestingly, the most evident functional effect was observed when the non-natural aa residue NAL was introduced at p4 of the peptide. Overall, such results suggest that different substitutions in p4 are able to break the immunological tolerance against tumor associated antigens and the elicited T cells are able to better control tumor growth.

In order to further improve the immunogenicity of peptides and reach the optimal affinity to the H-2Db molecule, a scaffold peptide was generated based on the consensus derived from the alignment of hundreds of H-2Db binding peptides. The most frequent amino acid residues found at p2, p3, p5 and p9 anchor positions were identified and the xALxNxxxL scaffold was used to present the TCR-facing residues of the HPV-E7 as well as the Trp2 peptides.

Bioinformatics structural analyses showed that the designed epitope scaffolds, regardless the amino acid residues at the TCR-facing positions, share the same conformation and contact pattern with the H-2Db groove, suggesting that a “universal” anchor structure for a given MHC molecule can be generated. Moreover, the conformation of the TCR-facing structure in the scaffold peptides did not show any appreciable difference with the corresponding wt, supporting that the “universal” scaffold can be used for different tumor antigens without modification of the peptide structure interacting with the TCR.

Such structural analysis was confirmed by a binding assay in the TAP-deficient RMA-S cell line, showing a significant increase in the affinity of the scaffolds compared to the corresponding wt peptide. In particular, such increase was striking for the Trp2 peptide whose affinity to the H-2Db molecule increased from 6990 to 19.6 nM (356.6 fold). A significant incremental effect (17.1 fold) was observed also for the HPV-E7 peptide even though the wt has already a very high affinity (99.7 nM).

Preclinical experiments in a therapeutic setting confirmed that scaffold peptides, with or without substitution in p4, are able to induce a cross-reactive T cell response able to control tumor growth at the same level of the wt peptide. As predictable, the efficiency was much higher for the TC1 than for the B16F10 “cold tumor” model (25). In particular, the latter experimental model confirms that the efficiency of a cancer vaccine strategy, even if based on an antigen with extremely high affinity to the MHC molecule and efficient presentation to the TCR, is strongly impaired by poor tumor T cell infiltration. Indeed, the intra-tumoral area of B16F10 tumors were infiltrated by low numbers of CD3^+^ as well as GrzB^+^ T cells. Furthermore, the GrzB^+^/FOXP3^+^ ratio confirms the immunosuppressive phenotype of Tumor microenvironment (TME) in the experimental groups. Also the increased number of effector T cells found in the wt scaff/p4mut group does not reach the density of infiltration reported in “hot tumors” (26) and the moderate positive GrzB^+^/FOXP3^+^ ratio is not sufficient to exert a significant anti-tumor effect. Moreover, also the significant increase of necrosis rate in the wt scaff/p4mut group is not is correlated with tumor regression.

In conclusion, the present study provides the experimental demonstration that heteroclitic peptides can be predicted and validated for eliciting a T cell response stronger than the corresponding wt peptide. Such response is cross-reactive with the wt peptide and is able to control tumor growth. Moreover, and even more importantly, it shows that a universal scaffold for a given MHC molecule can be predicted and validated for the optimal presentation of different antigen sequences to TCR. Such “MHC-optimized” antigens can be of high clinical relevance in cancer vaccine development.

## Data Availability Statement

The original contributions presented in the study are publicly available. This data can be found here: https://doi.org/10.5281/zenodo.5552505.

## Ethics Statement

The animal study was reviewed and approved by National Cancer Institute IRCCS Pascale.

## Author Contributions

CR and CM performed all the epitope conformation and *in vitro* validation. BC performed bioinformatics predictions of binding affinity and *in vitro* validation. AL, AB, and GP performed the *in vivo* immunizations. AM performed the immunological analysis on animal samples. GS, AM, and MB analyzed the tumor tissues by pathology IHC. LV contributed to the conformational analyses. AC performed the peptide synthesis, and MR supervised the optimization of the peptide synthesis. MLT and FB contributed to data analysis. MT and LB designed the study, supervised the analysis and drafted the manuscript. All authors read and approved the final manuscript.

## Funding

The study was funded by Italian Ministry of Health through Institutional “Ricerca Corrente” (LB). POR FESR 2014/2020 “Campania OncoTerapie” (LB, LV, and MR). AM is funded by “Ricerca Corrente”. CM, BC, and CR are funded by POR FESR 2014/2020 “NanoCAN”.

## Conflict of Interest

The authors declare that the research was conducted in the absence of any commercial or financial relationships that could be construed as a potential conflict of interest.

## Publisher’s Note

All claims expressed in this article are solely those of the authors and do not necessarily represent those of their affiliated organizations, or those of the publisher, the editors and the reviewers. Any product that may be evaluated in this article, or claim that may be made by its manufacturer, is not guaranteed or endorsed by the publisher.

## Supplementary Material

Supplementary Figure 1 Therapeutic immunization protocol. C57BL/6 mice were subcutaneously injected with 5x10^4^ and 1x10^5^ cells/mouse of B16F10 or TC1 respectively on the right back flank. When the tumor diameter reached 4-6 mm, mice were randomly divided into six groups and treated according to the scheme. The tumor size was measured and documented every two days with a caliper, starting on day 7 and animals were sacrificed when tumor volume was greater than 1600 mm3

Supplementary Figure 2 Effect on tumor growth of therapeutic immunization with htcPep in TC1 tumor model. C57BL/6 mice were administered with 1x10^5^ cells/mouse of TC1. Tumor growth was evaluated every three days with a caliper and tumor volume was calculated as indicated in Materials and Methods.

Supplementary Figure 3 Effect on tumor growth of therapeutic immunization with htcPep in B16F10 tumor model. C57BL/6 mice were administered with 5x10^4^ cells/mouse of B16F10. Tumor growth was evaluated every three days with a caliper and tumor volume was calculated as indicated in Materials and Methods.

Supplementary Figure 4 Logo plot showing sequence analysis of all 9-mer H-2Db peptides. A total number of 250 predicted strong binders were aligned from position 1 to position 9 and analyzed by the online Seq2Logo - 2.0.

Supplementary Figure 5 Epitope scaffold sequences. E7 scaffold sequences **(A)** Trp2 Scaffold sequences **(B)**. The green boxes indicate the anchor positions. The predicted binding affinity to H2-Db molecule of each peptide for each peptide are shown **(C, D)**.

Supplementary Figure 6
*In vitro* analysis of scaffold binding affinity to H-2Db molecule. Binding to H2-Db molecule was assessed in TAP-deficient RMA-S murine cells loaded with 10 μM the indicated peptides. Mean fluorescence intensity at flow cytometer indicates binding levels of each peptide to the H2-Db. Fold-increase (wt = 1) of the binding to the H2-Db molecule of each peptide. Overlay of the mean fluorescence intensity observed with different peptides.

Supplementary Figure 7 Therapeutic immunization protocol with epitope scaffolds. C57BL/6 mice were subcutaneously injected with 5x10^4^ and 1x10^5^ cells/mouse of B16F10 or TC1 respectively on the right back flank. When the tumor diameter reached 4-6 mm, mice were randomly divided into five groups and treated according to the scheme. The tumor size was measured and documented every two days with a caliper, starting on day 7 and animals were sacrificed when tumor volume was greater than 1600 mm^3^

Supplementary Figure 8 IHC staining. Representative images of IHC staining of CD3^+^, FoxP3^+^ and GranzymeB^+^ cells in the Core-Tumor (CT) and Invasive Margin (IM) areas in the WT **(A, C, E)** and WT scaff/mut **(B, D, F)** groups. Positive cells are indicated by intense red color.

Supplementary Figure 9 Images of tumor necrosis rates. Representative H&E stain showing tumor necrosis in each experimental groups. Necrotic areas are indicated by lighter color.
