# Supplementary figures and images for: MHC-Optimized Peptide Scaffold for Improved Antigen Presentation and Anti-Tumor Response
Source: Front Immunol. 2021 Oct 20;12:769799. doi: 10.3389/fimmu.2021.769799 (PMC8564487; doi:10.3389/fimmu.2021.769799)

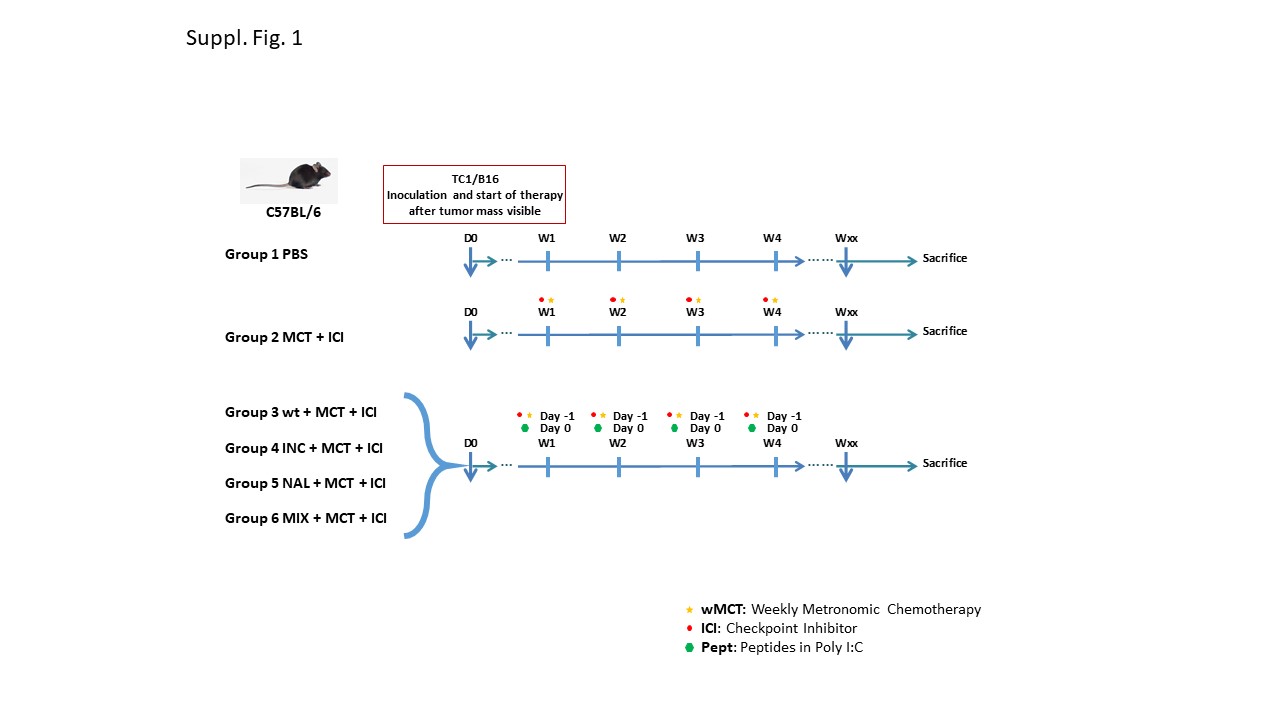

Supplement: Supplementary Figure 1 — Therapeutic immunization protocol. C57BL/6 mice were subcutaneously injected with 5x10^4^ and 1x10^5^ cells/mouse of B16F10 or TC1 respectively on the right back flank. When the tumor diameter reached 4-6 mm, mice were randomly divided into six groups and treated according to the scheme. The tumor size was measured and documented every two days with a caliper, starting on day 7 and animals were sacrificed when tumor volume was greater than 1600 mm3 [file Image_1.jpeg]

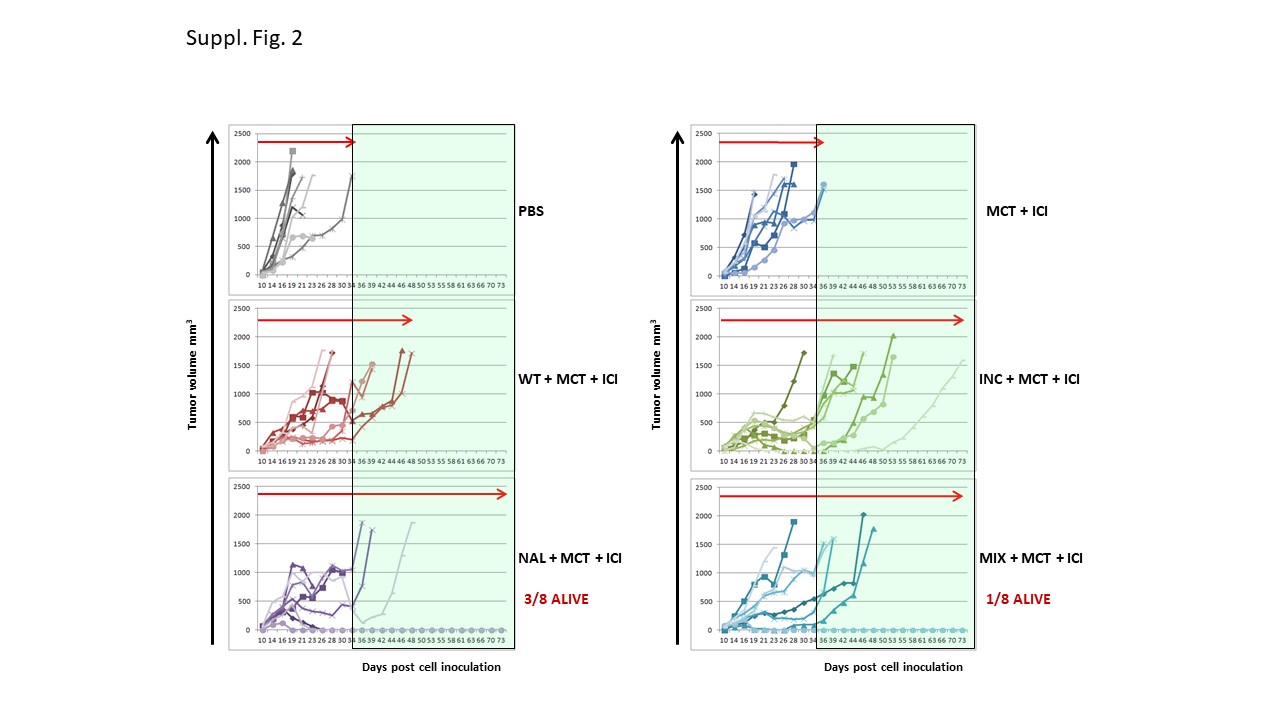

Supplement: Supplementary Figure 2 — Effect on tumor growth of therapeutic immunization with htcPep in TC1 tumor model. C57BL/6 mice were administered with 1x10^5^ cells/mouse of TC1. Tumor growth was evaluated every three days with a caliper and tumor volume was calculated as indicated in Materials and Methods. [file Image_2.jpeg]

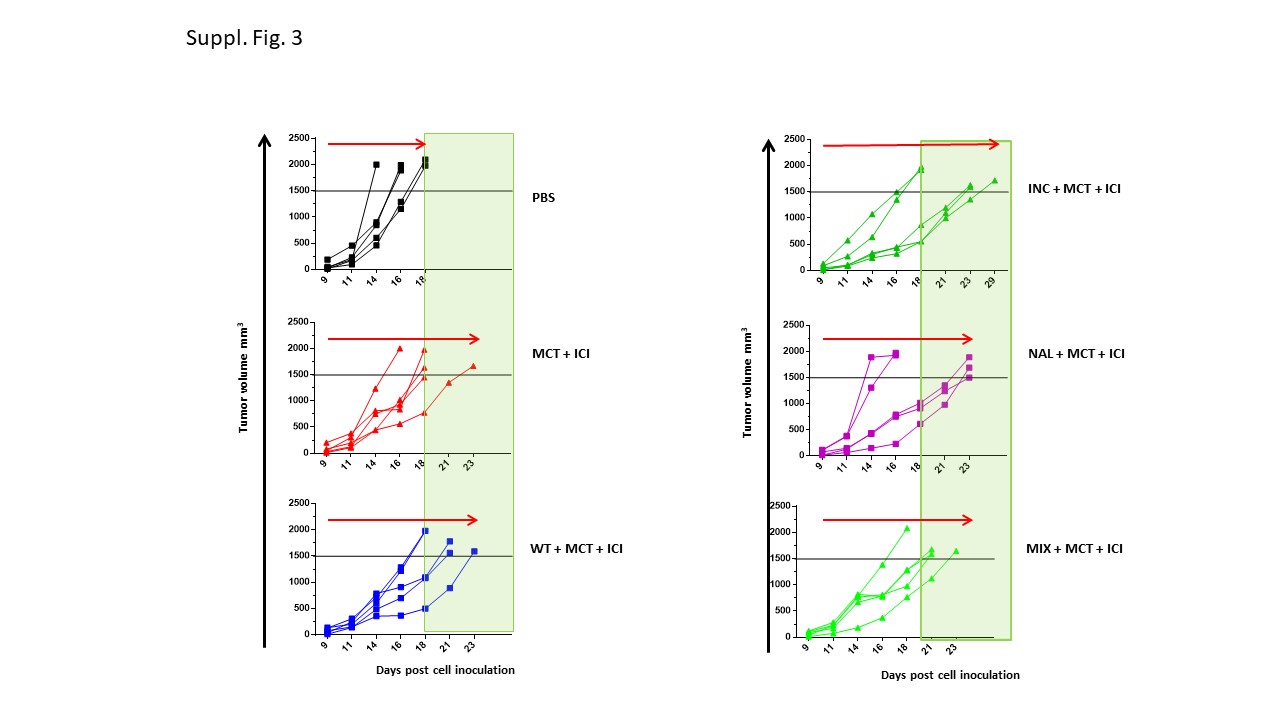

Supplement: Supplementary Figure 3 — Effect on tumor growth of therapeutic immunization with htcPep in B16F10 tumor model. C57BL/6 mice were administered with 5x10^4^ cells/mouse of B16F10. Tumor growth was evaluated every three days with a caliper and tumor volume was calculated as indicated in Materials and Methods. [file Image_3.jpeg]

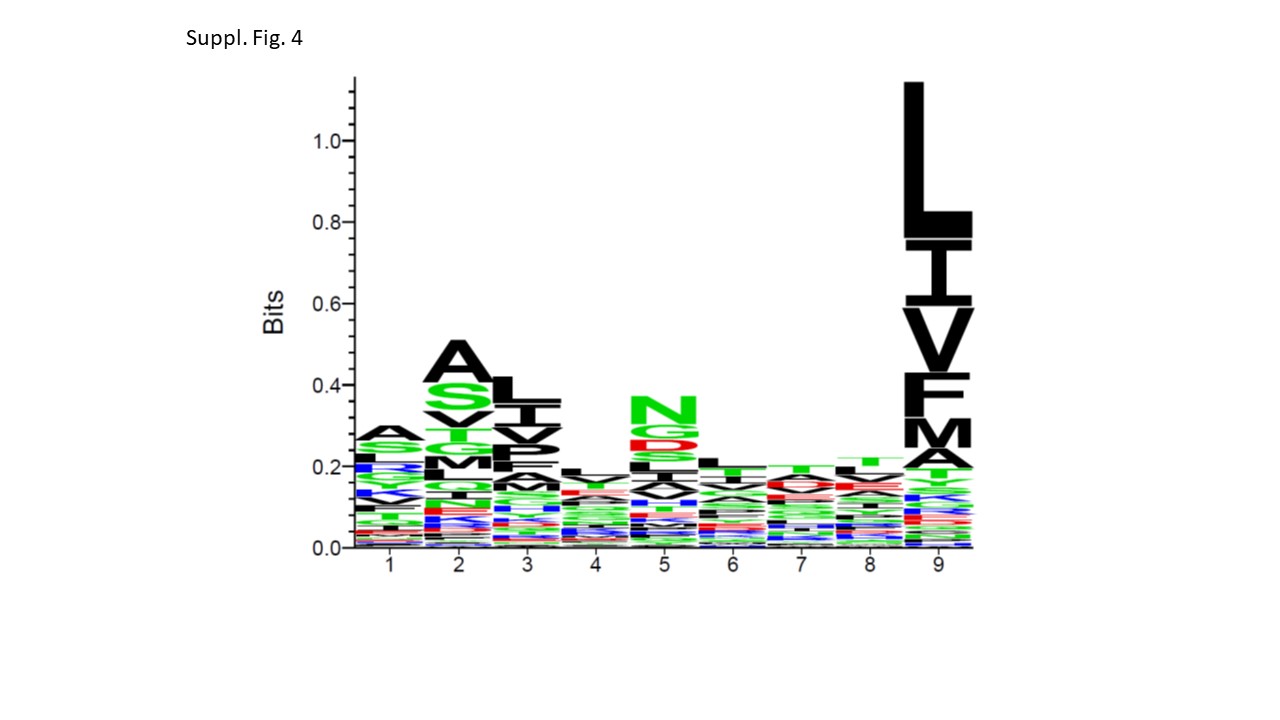

Supplement: Supplementary Figure 4 — Logo plot showing sequence analysis of all 9-mer H-2Db peptides. A total number of 250 predicted strong binders were aligned from position 1 to position 9 and analyzed by the online Seq2Logo - 2.0. [file Image_4.jpeg]

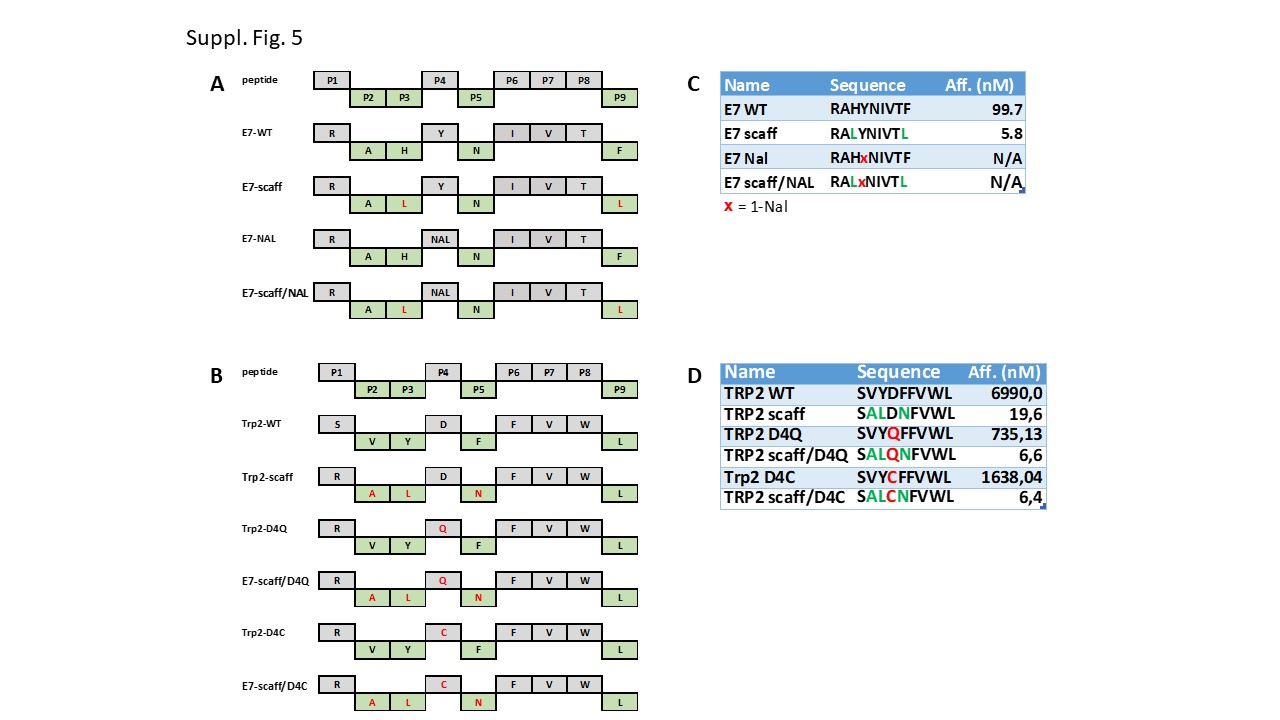

Supplement: Supplementary Figure 5 — Epitope scaffold sequences. E7 scaffold sequences (A) Trp2 Scaffold sequences (B). The green boxes indicate the anchor positions. The predicted binding affinity to H2-Db molecule of each peptide for each peptide are shown (C, D). [file Image_5.jpeg]

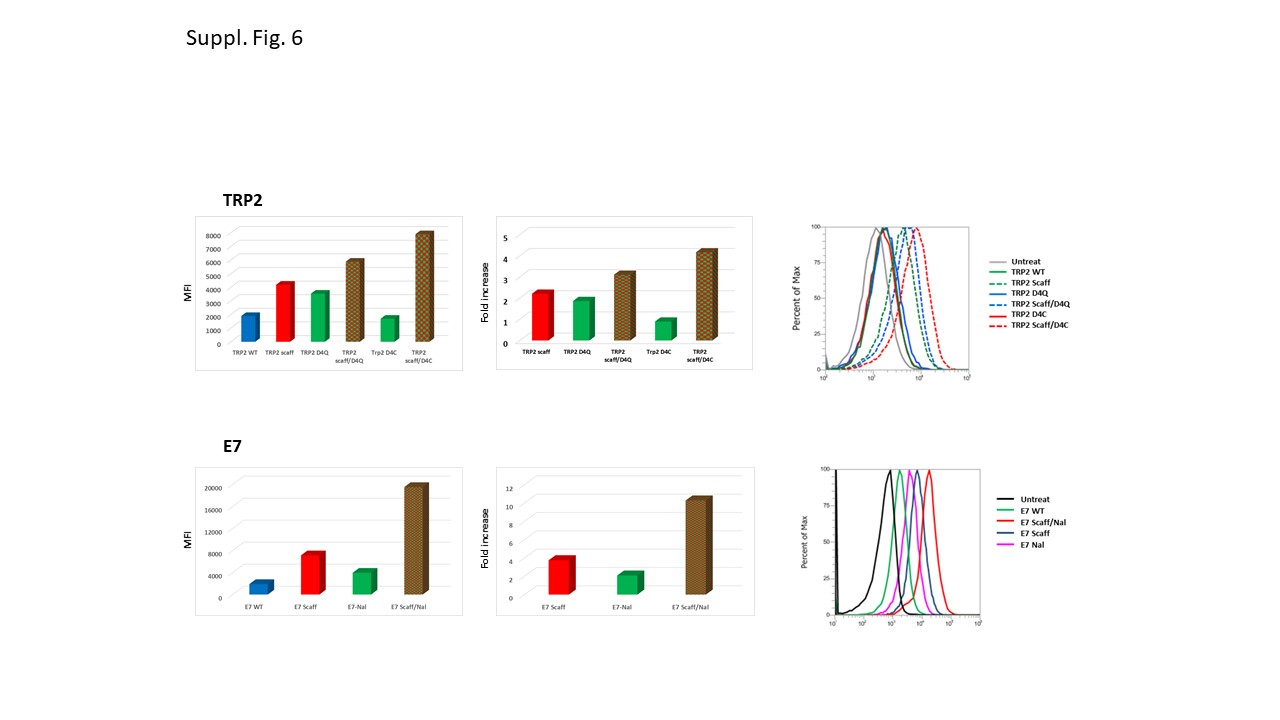

Supplement: Supplementary Figure 6 — In vitro analysis of scaffold binding affinity to H-2Db molecule. Binding to H2-Db molecule was assessed in TAP-deficient RMA-S murine cells loaded with 10 μM the indicated peptides. Mean fluorescence intensity at flow cytometer indicates binding levels of each peptide to the H2-Db. Fold-increase (wt = 1) of the binding to the H2-Db molecule of each peptide. Overlay of the mean fluorescence intensity observed with different peptides. [file Image_6.jpeg]

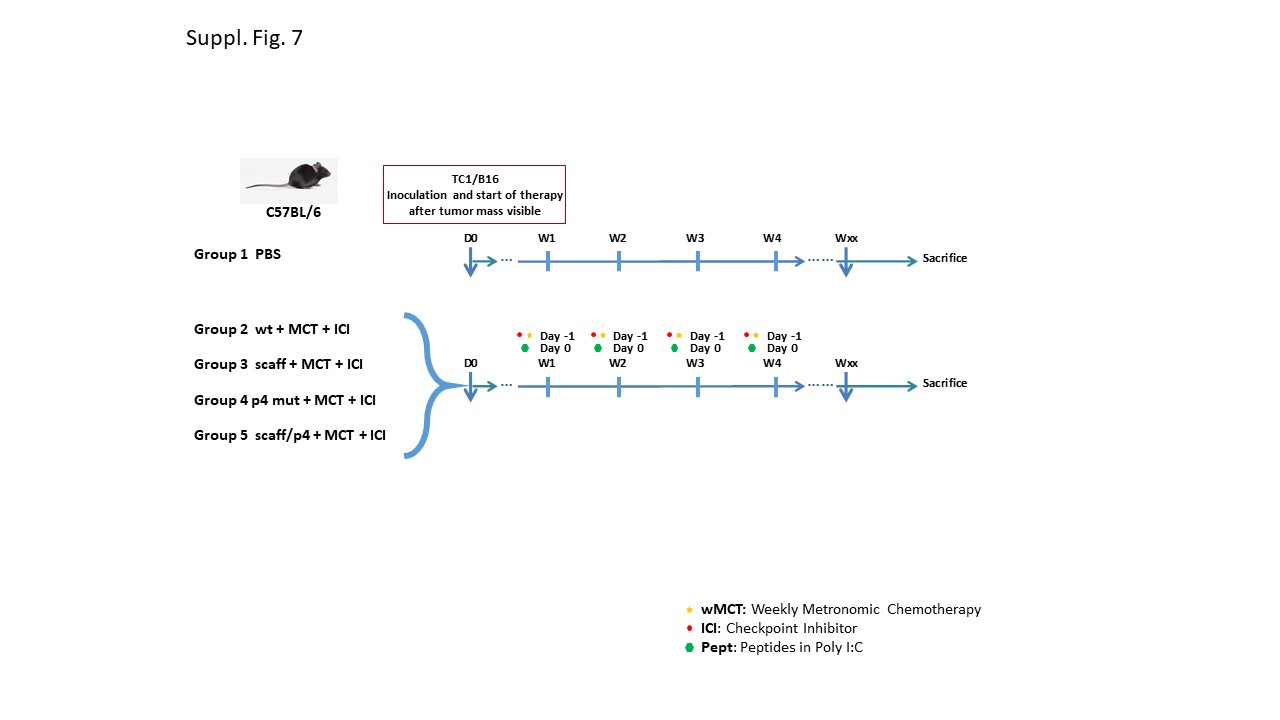

Supplement: Supplementary Figure 7 — Therapeutic immunization protocol with epitope scaffolds. C57BL/6 mice were subcutaneously injected with 5x10^4^ and 1x10^5^ cells/mouse of B16F10 or TC1 respectively on the right back flank. When the tumor diameter reached 4-6 mm, mice were randomly divided into five groups and treated according to the scheme. The tumor size was measured and documented every two days with a caliper, starting on day 7 and animals were sacrificed when tumor volume was greater than 1600 mm3 [file Image_7.jpeg]

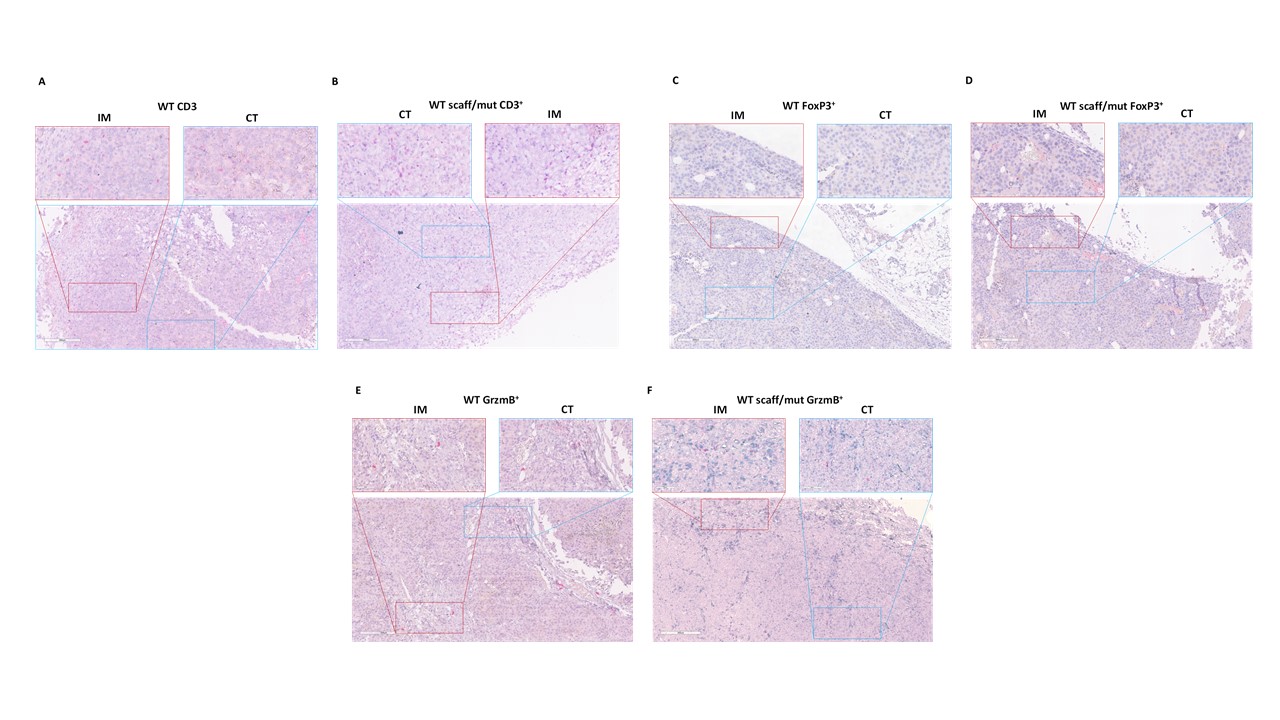

Supplement: Supplementary Figure 8 — IHC staining. Representative images of IHC staining of CD3+, FoxP3+ and GranzymeB+ cells in the Core-Tumor (CT) and Invasive Margin (IM) areas in the WT (A, C, E) and WT scaff/mut (B, D, F) groups. Positive cells are indicated by intense red color. [file Image_8.jpg]

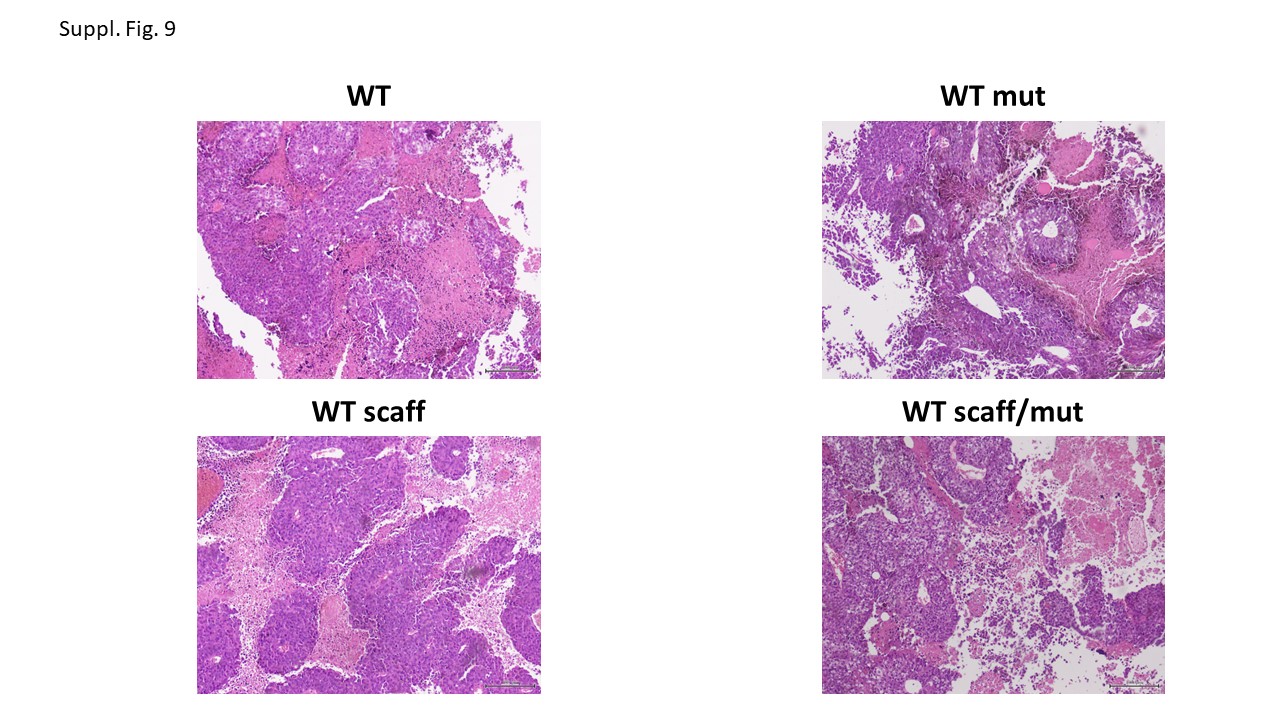

Supplement: Supplementary Figure 9 — Images of tumor necrosis rates. Representative H&E stain showing tumor necrosis in each experimental groups. Necrotic areas are indicated by lighter color. [file Image_9.jpeg]
